# Erratum: Evolution of structure functions in momentum space

**DOI:** 10.1140/epjc/s10052-025-13830-w

**Published:** 2025-01-27

**Authors:** Tuomas Lappi, Heikki Mäntysaari, Hannu Paukkunen, Mirja Tevio

**Affiliations:** 1 University of Jyvaskyla, Department of Physics, P.O. Box 35, FI-40014 University of Jyvaskyla, Finland; 2 Helsinki Institute of Physics, P.O. Box 64, FI-00014 University of Helsinki, Finland

**Erratum: Eur. Phys. J. C (2024) 84:84** 10.1140/epjc/s10052-023-12365-2

This erratum corrects few typos in the article.

The term $N_\textrm{c}\frac{b_0}{3}$ is corrected to $b_0$ in Eqs. (40) and (68).

Equation (41) has been updated to

1 $$\begin{aligned} P^{2\text {-}o.}_{F_{\textrm{L}}\widetilde{F}_{\textrm{L}}}(z)&\equiv \left[ \frac{C_\textrm{F}}{2} +b_0 \right] \delta (1-z) +\Bigg [C_\textrm{F}-2N_\textrm{c}+2N_\textrm{c}\frac{1}{z}\nonumber \\&\quad +(4N_\textrm{c}-C_\textrm{F})z+4N_\textrm{c}z^2\log \left( 1-z\right) \Bigg ] . \end{aligned}$$

Equation (42) has been updated to

2 $$\begin{aligned} P^{2\text {-}o.}_{F_{\textrm{L}}\widetilde{F'}_{\textrm{L}}}(z) \equiv -2N_\textrm{c}z^2\log \left( 1-z\right) . \end{aligned}$$

Equation (64) has been updated to

3 $$\begin{aligned} P_{qg}\otimes g&= \frac{1}{4n_\textrm{f} \bar{e}_q^2}\Bigg \{C_\textrm{F}\Bigg [-\widetilde{F}_2 (x, Q^2)\nonumber \\&\quad +2\int _x^1\frac{\hbox {d}\xi }{\xi }\left( \frac{x}{\xi }-1\right) \widetilde{F}_2(\xi , Q^2)\Bigg ] \nonumber \\&\quad +\frac{1}{2}\Bigg [ \widetilde{F}_{\textrm{L}} (x, Q^2)-\widetilde{F'}_{\textrm{L}} (x, Q^2)\nonumber \\&\quad +2\int _x^1\frac{\hbox {d}{\xi }}{\xi }\widetilde{F}_{\textrm{L}}(\xi , Q^2)\Bigg ]\Bigg \}. \end{aligned}$$

